# Neuroinflammation of Microglial Regulation in Alzheimer’s Disease: Therapeutic Approaches

**DOI:** 10.3390/molecules29071478

**Published:** 2024-03-26

**Authors:** Haiyun Chen, Yuhan Zeng, Dan Wang, Yichen Li, Jieyu Xing, Yuejia Zeng, Zheng Liu, Xinhua Zhou, Hui Fan

**Affiliations:** 1 College of Pharmacy, Clinical Pharmacy (School of Integrative Pharmacy), Guangdong Pharmaceutical University, Guangzhou 510006, China; yx07230chy@126.com (H.C.);; 2 Guangdong Metabolic Diseases Research Center of Integrated Chinese and Western Medicine, Guangzhou 510006, China; ted20192022@outlook.com (Y.Z.);; 3 Guangdong TCM Key Laboratory for Metabolic Diseases, Guangzhou 510006, China; 4 Key Laboratory of Glucolipid Metabolic Disorder, Ministry of Education of China, Guangzhou 510006, China; 5 Key Unit of Modulating Liver to Treat Hyperlipemia SATCM, State Administration of Traditional Chinese Medicine, Guangzhou 510006, China; 6 Guangdong Provincial Key Laboratory of Research and Development of Natural Drugs, School of Pharmacy, Guangdong Medical University, Zhanjiang 524023, China; lycoco2021@163.com; 7 School of Medicine, Foshan University, Foshan 528000, China; liuzheng8709@fosu.edu.cn; 8 Guangzhou Eighth People’s Hospital, Guangzhou Medical University, Guangzhou 510000, China

**Keywords:** Alzheimer’s disease, neuroinflammation, microglial activation, anti-inflammatory agents, microglial energy metabolic disorder, microglial regulation

## Abstract

Alzheimer’s disease (AD) is a complex degenerative disease of the central nervous system that is clinically characterized by a progressive decline in memory and cognitive function. The pathogenesis of AD is intricate and not yet fully understood. Neuroinflammation, particularly microglial activation-mediated neuroinflammation, is believed to play a crucial role in increasing the risk, triggering the onset, and hastening the progression of AD. Modulating microglial activation and regulating microglial energy metabolic disorder are seen as promising strategies to intervene in AD. The application of anti-inflammatory drugs and the targeting of microglia for the prevention and treatment of AD has emerged as a new area of research interest. This article provides a comprehensive review of the role of neuroinflammation of microglial regulation in the development of AD, exploring the connection between microglial energy metabolic disorder, neuroinflammation, and AD development. Additionally, the advancements in anti-inflammatory and microglia-regulating therapies for AD are discussed.

## 1. Introduction

Alzheimer’s disease (AD) is a complex neurodegenerative order that primarily affects memory and cognitive function. It is the most common cause of dementia, accounting for 60–80% of cases. According to the World Alzheimer Report 2019, the prevalence of AD dementia is projected to rise from 50 million globally in 2019 to 152 million by 2050 due to the aging population [1]. This translates to a new dementia case every three seconds and an estimated annual cost of USD 1 trillion, which is expected to double by 2030. Given the increasing number of AD patients and the rising costs associated with their care, there is a pressing need to develop effective treatments for this condition.

The pathological features of AD are primarily characterized by the formation of senile plaques (SPs) through amyloid beta (Aβ) aggregation and neurofibrillary tangles (NFTs) due to tau protein hyperphosphorylation [2]. Research indicates that many neurodegenerative diseases stem from the accumulation of misfold proteins, such as Aβ aggregation and hyperphosphorylated tau in AD, as well as α-synuclein accumulation in Parkinson’s disease (PD) [3]. Targeting these misfolded proteins to slow down or delay the progression of neurodegenerative disorder has been a significant focus in drug development. Over 200 anti-AD drugs targeting Aβ and tau therapies have undergone clinical trials, but only a few have received approval from the Food and Drug Administration (FDA) with considerable controversy [4], highlighting the need for a better understanding of the pathological mechanism of AD. Recent studies have revealed the complexity of AD pathogenesis, involving factors like Aβ, tau, neuroinflammation, and the immune system, forming an intricate network that regulates AD pathology [5]. Among them, microglial activation can enhance Aβ clearance [6] and directly drive the spread of tau proteins in the Braak phase, and even Aβ-mediated tau spread is also dependent on microglial activation [7], indicating the pivotal role of microglial activation-mediated neuroinflammation in AD risk, onset, and advancement [8]. Such microglial activation is also closely linked to metabolic inflammation in the body, leading to a growing interest in the use of anti-inflammatory drugs for AD prevention and treatment [9]. Therefore, we present a review of the role of microglial activation-mediated neuroinflammation and microglial energy metabolic disorder in the pathological process of AD, as well as the potential therapeutic interventions through small molecule drug treatments.

As reported, Aβ deposition is key factor in the pathogenesis of AD, resulting from the cleavage of amyloid precursor protein (APP) by secretase enzymes, mainly including alpha-secretase, β-secretase, and gamma-secretase [10]. These enzymes generate soluble APPα, APPβ, Aβ, and APP intracellular domains [11,12]. While soluble peptides can be recycled intracellularly, Aβ peptides can be further cleaved into shorter peptides like Aβ40 and Aβ42 [13]. Aβ42 is particularly prone to forming beta-amyloid plaques (ABPs), considered critical in AD pathogenesis. ABPs may disrupt the signaling between neurons, leading to brain damage, memory loss, and recognition issues [14]. ABPs have also been reported to initiate an immune response that leads to neuroinflammation, which may damage the surrounding neurons [15]. Additionally, ABPs trigger an immune response causing neuroinflammation that harms surrounding neurons. ABPs are also found in cerebral amyloid angiopathy (CAA) associated with AD [16]. CAA can eventually result in the leakage or rupture of blood vessels located outside of the cell. Another neuropathological characteristic of AD is the presence of NFTs, which are aggregates of abnormal tau proteins found in the neuronal cytoplasm that form paired helical filaments [17]. Normally, tau proteins are situated on the surface of microtubules and contribute to their structural integrity. When the formation of ABPs in the extracellular region occurs, ABPs can transfer a phosphate group to the tau protein through the activation of the kinases pathway, resulting in the phosphorylated tau protein becoming isolated from the microtubule [18]. Such phosphorylated tau proteins can make microtubules incomplete, leading to a loss of their signaling function, cell death, or apoptosis [19]. Given the crucial roles of Aβ and tau in the pathogenesis of AD, over 200 anti-AD drugs targeting these proteins have been tested in clinical trials to slow down disease progression, although only a few have received FDA approval [2]. The Aβ and tau hypothesis of AD is under scrutiny, and the development of disease-modifying treatments for AD has proven to be challenging. In recent decades, neuroinflammation has emerged as a third major neuropathological feature in the brains of AD patients, alongside Aβ and NFTs, forming a complex network that influences AD pathology [20,21,22,23]. Increased pro-inflammatory cytokines, such as tumor necrosis factor α (TNF-α) and interleukin 6 (IL-6), have been found in the serum and brain tissue of individuals with AD when compared to controls [24]. The initial phases of AD are marked by persistent neuroinflammation. This early inflammation, as the disease progresses, contributes to and intensifies the production of Aβ and NFT, leading to neuronal toxicity and death [25,26,27]. Additionally, endogenous bioactive lipids including eicosanoids, specialized pro-catabolic lipid mediators, lysophospholipids, and endocannabinoids in the brain which regulate a multitude of cellular and molecular processes, are closely linked to chronic inflammation [28]. The disruption of these bioactive lipids significantly enhances the development and progression of neurodegenerative diseases, leading to chronic damage like AD [10]. Ongoing inflammation leads to the continuous release of different inflammatory cytokines, resulting in a pro-inflammatory response that outweighs the anti-inflammatory response, and ultimately harming neurons and causing various pathological changes in the body [29,30,31]. Epidemiological studies have shown that the delayed onset of AD in certain populations due to the use of anti-inflammatory drugs implies a potential role in modulating neuroinflammation [32], underscoring the significance of neuroinflammation in the development of neurodegenerative diseases.

## 2. Microglia’s Role in AD

### 2.1. Main Physiological Functions of Microglia

The central nervous system (CNS) is a complex network of neurons and glial cells, of which glial cells are composed of astrocytes, oligodendrocytes, and NG2 cells composed of macroglia and microglia. These cells provide crucial support for brain functions. Microglia, a type of macrophage, are innate immune cells in the CNS that originate from a single mesoderm but have multiple neuroectodermal lineages, distinguishing them from other brain cells. Microglia play key roles in various physiological processes such as brain development, activity, and plasticity [33]. As reported, microglia are crucial regulators of CNS development and homeostasis through neuron–microglial interactions, removal of cellular debris, secretion of trophic factors, and synaptic pruning and remodeling [34]. Additionally, microglia also contribute to memory and cognitive functions by modulation of neuronal numbers and neural networks [35]. Furthermore, they support neurogenesis and neuronal survival, promoting circuit plasticity, as well as neuronal survival by supporting trophic factors, suggesting neuron–glia interaction is important for network formation in the developing brain [36,37,38]. Importantly, microglia can also accumulate in the cerebellar region to eliminate the dying neurons and nonfunctional synapses with high levels of basal clearance activity [39]. As the primary innate immune cells in the CNS, microglia respond to harmful stimuli through recognition of pathogen-associated molecular patterns (PAMPs) and damage-associated molecular patterns (DAMPs) via receptors like pattern recognition receptors (PRRs) and viral receptors [40]. Concretely, PAMPs are typically shared structures of pathogens, which can be recognized by lipopolysaccharide (LPS) in bacterial cell walls and double-stranded RNA in viruses through PRRs including Toll-like receptors (TLRs) and NOD-like receptors (NLRs) activating immune cells and promoting inflammatory responses, whereas DAMPs are endogenous molecules released during cell damage or death, which can also be recognized by PRRs and trigger immune responses [41]. The recognition and immune responses to PAMPs and DAMPs play a crucial role in the immune system’s defense against external and internal threats [42,43]. In response to these insults, microglia can be activated and then secrete pro-inflammatory and anti-inflammatory factors, contributing to a double-edged sword role in CNS damage [44,45].

### 2.2. Microglial Activation-Mediated Neuroinflammation in AD

Under physiological conditions, microglia may appear to be in a ‘resting’ state, but they are actually extending their cell bodies to continually monitor potential brain damage [46,47]. In situations of pathological injury, microglia are rapidly activated and polarized, exhibiting processes such as proliferation, chemotaxis, phagocytosis, migration, and cytokine secretion. Depending on the severity of the damage, if the damage is mild, microglia quickly transition to anti-inflammatory ‘M2’ phenotypes, characterized by distant branching, minor cell body alterations, and release of anti-inflammatory factors. ‘M2’ microglia contribute to tissue repair, phagocytic activity enhancement, and neuroprotective effects. In cases of severe or prolonged damage, cells at the injury site emit signals to be phagocytized. Subsequently, microglia become fully activated, polarizing to pro-inflammatory ‘M1’ phenotypes or the toxic ‘M1’ state with round cell bodies and thickened protrusions [48,49,50]. These microglial cells mainly exert cytotoxic effects with the capability of efficiently clearing infected cells and debris from dying cells [50]. However, excessive activation of ‘M1’ microglia can result in neuronal function loss, damage, and degeneration, playing crucial roles in cerebrovascular and neurodegenerative diseases [49,51]. In the context of normal pathological damage, due to the diverse signals emitted by cells within the organism, microglial cells may polarize into either ‘M1’ phenotype or ‘M2’ phenotype states to protect the nervous system [50]. Overall, there is a strong controversy regarding this two-phenotype classification by simply defining their activated states since this method is not applicable for microglial cells in a complex brain environment. Commonly, over-activation of the ‘M1’ phenotype can harm neuronal function in AD [50,52,53]. It is well known that the anti-inflammatory phenotype microglia confer neuroprotective effects by increasing the expression of cytokines and proteins including the arginase 1 gene (Arg-1), transforming growth factor-β (TGF-β), and interleukin 10 (IL-10) involved in resolving inflammation, maintaining homeostasis, and promoting wound healing [54,55,56]. However, this initial anti-inflammatory response is self-limiting [57], since neuroinflammation occurring in neurodegenerative disease tends to be a chronic process, characterized by long-term stimuli-induced over-activation that can surpass the self-limiting capacity of the organism. Such over-activation leads to microglia continuously transforming into pro-inflammatory phenotypes and releasing significant quantities of pro-inflammatory factors such as TNF-α, interleukin 1β (IL-1β), and IL-6. These pro-inflammatory factors lead to neurotoxic responses such as oxidative stress and neuronal apoptosis, which are key triggers for the development of neurodegenerative diseases like AD [58]. This can also be used to explain the different phenotypes of activated microglia in the AD brain. Initially, microglia effectively remove excessive Aβ and tau through phagocytosis at the onset of neuroinflammation. However, prolonged microglial over-activation can lead to Aβ and tau aggregation, accelerating the progression of AD [59]. In particular, a large accumulation of Aβ can hinder microglial phagocytosis by reducing the activity of phagocytic receptors on microglia, increasing pro-inflammatory cytokines production and further Aβ accumulation. This forms a vicious positive feedback loop that accelerates neurodegeneration and neuronal death [60].

The modulation of microglia on tau involves the ability of microglia to remove the neurons containing tau protein, secrete tau protein, and then subsequently transmit tau protein to other neurons [61]. The secreted tau protein acts as “tau seeds”, leading to the accumulation of tau protein in the recipient cells [62,63,64]. However, the release of a large misfolded protein into the extracellular space may also lead to a role for microglia in clearance or processing of tau, since microglia are the obligate phagocytes of the brain. Microglia are capable of degrading tau protein, but their efficiency in this process is not as expected. The ineffective processing of tau protein by microglia could potentially contribute to the spread of tau pathology in vivo [65]. The continuous accumulation of tau in brain regions, along with the induction of phosphorylated tau protein by extracellular tau seeds, contributes to a vicious cycle. Additionally, neuroinflammation has been reported to exacerbate the pathological progression of tau by disrupting neural transport and inhibiting mitochondrial respiration [3]. It has been demonstrated that injurious stimuli such as LPS, prostaglandin E2, and tert-butyl hydroperoxide can trigger over-activation of microglia, leading to the promotion of Aβ aggregation [66]. Among them, LPS can also induce phosphorylation of tau protein in rTg4510 mice, resulting in the formation of NFTs [67], and this effect is associated with the activation of cyclin-dependent kinase 5 (CDK5) [68]. In vivo experiments utilizing gene silencing to induce microglial activation have revealed increased tau protein phosphorylation in the hippocampus and impaired synaptic integrity, highlighting the significant role of microglial activation-mediated neuroinflammation in the pathological progression of AD [69].

## 3. The Critical Role of Energy Metabolic Disorder of Microglia between Microglial Activation-Mediated Neuroinflammation and AD

The current evidence indicates that chronic peripheral inflammation could potentially initiate systemic inflammation, increasing production of pro-inflammatory cytokines and other mediators, which in turn trigger neuroinflammation in the diseased brain [70,71]. In instances of systemic metabolic inflammation, pro-inflammatory mediators can enter the brain, inducing microglial phagocytosis to engulf cell debris and damaged neurons by prompting microglia to swiftly alter their shape and extend their synapses [72]. The above morphological changes, phagocytosis, and translocation of microglial cells all require dynamic reorganization of the actin cytoskeleton, thus demanding a significant amount of adenosine triphosphate (ATP) support [73]. As the systemic metabolic inflammation-induced damage worsens, the insufficient ATP supply makes microglial cells transition from oxidative phosphorylation (OXPHOS) to frequent glycolysis to enhance ATP production [74]. As reported, in the context of neuroinflammation, pro-inflammatory microglial cells exhibit heightened glucose uptake and increased expression of glycolytic enzymes like hexokinase (HK), phosphofructokinase (PFK), and pyruvate kinase M2 (PKM2) [75]. When stimulated by LPS, microglial cells demonstrate elevated levels of glucose transporter 1 (GLUT1), HK2, and PFK1, indicating enhanced glycolytic capacity [76]. Conversely, blocking GLUT1 or inhibiting HK2 activity can diminish the production of pro-inflammatory cytokines, underscoring the critical role of glycolysis in driving the inflammatory response [77,78]. Recent studies have reported that the key glycolytic enzyme PKM2 also has a role in regulating pro-inflammatory microglial cell activation [78]. Nuclear PKM2 acts as a coactivator of the transcription factor STAT1, which promotes the expression of pro-inflammatory genes. Silencing PKM2 with TEPP-46 or inhibiting its nuclear translocation significantly reduces the inflammatory response in microglial cells [79]. Additionally, lactate, a product of glycolysis, has been found to enhance the release of pro-inflammatory factors by microglial cells [80] and induce histone lactylation, impacting macrophage polarization [81]. Studies have shown that pan-protein lysine lactylation and H3K18 lactylation are upregulated in aging microglial cells and hippocampal tissues of naturally aged mice and AD model mice [82], suggesting a crucial role of lactylation in glycolysis in the progression of AD. Conversely, sustained activation of microglial cells may increase the expression of genes related to glycolysis, promoting glycolysis and exacerbating neurodegenerative diseases like AD and PD [83,84,85]. Ruiyuan Pan’s research on AD revealed that excessive glucose uptake by microglial cells results in the production of high levels of lactate through glycolysis. This lactate induces lactylation epigenetic modifications on histones, which then regulate glycolytic genes like PKM2 to establish a glycolysis/H4K12 lactylation/PKM2 positive feedback loop. Such loop formation then promotes the build-up of significant inflammation, ultimately contributing to the pathology of AD [86]. Based on the crucial role of glycolysis in the activation and polarization of microglial cells during neuroinflammation, targeting related glycolytic enzymes or pathways during glycolysis period would be new therapeutic approaches to mitigate microglial activation-mediated neuroinflammation.

**Figure 1:**
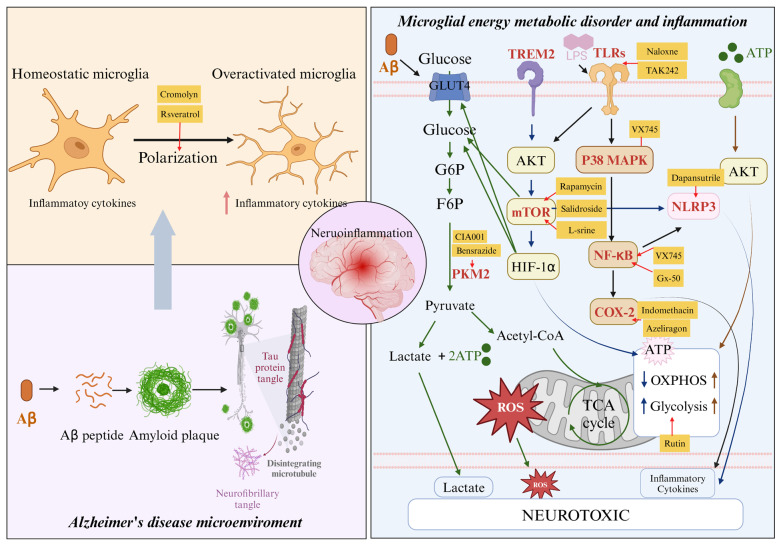
Energy metabolic disorder and inflammation of microglia in AD in response to therapy. When exposed to the AD environment, microglia polarizing to pro-inflammatory ‘M1’ phenotypes release many inflammatory cytokines. Microglia activate TLRs, which, in turn activate the TREM2/AKT/mTOR signaling pathway, leading to the upregulation of HIF-1α, GLUT, and PKM2. This results in reduced OXPHOS, leading to the production of reactive oxygen species (ROS) and inflammatory factors. Furthermore, the TREM2/AKT/mTOR and TLR4/NF-κB signaling pathways directly activate NLRP3, promoting inflammatory gene expression and further enhancing glycolysis. The drugs indicated in yellow text boxes act through target genes (in red) to induce anti-inflammatory responses and regulate microglial polarization and energy metabolic disorder, ultimately aiming to alleviate or treat AD.

Current research suggested that the body undergoing frequent glycolysis decreased the microglial phagocytosis and migration activity for Aβ clearance [87]. The accumulation of Aβ could also impede glucose uptake, aerobic glycolysis, and ATP synthesis by regulating glucose transporter 4 (GLUT4) and phosphofructokinase. Studies have demonstrated a correlation between the glycolysis transition and the reduced ability of microglia to phagocytose Aβ [88]. As reported, the triggering receptor expressed on myeloid cells 2 (TREM2) found on microglia plays a vital role in Aβ phagocytosis [89,90], while the absence of TREM2 increased the accumulation of Aβ plaques [91]. Further analysis of mice lacking TREM2 reported that they displayed AD-like symptoms and exhibited reduced glycolysis and relative difficulty in clearing Aβ [92]. Besides the crucial role of TREM2 in altering microglial metabolism, inflammasomes, particularly the NLR family pyrin domain-containing 3 (NLRP3) inflammasome, are also implicated in AD pathology (Figure 1) [93]. The NLRP3 inflammasome serves as a sensor of the innate immune system and can be triggered by various factors, including Aβ and oxidative stress [94]. Activation of the NLRP3 inflammasome in microglia results in the release of pro-inflammatory cytokines like IL-1β and IL-18, leading to heightened neuroinflammation and neuronal damage [95]. The energy metabolic disorder of microglia is linked to the activation of the NLRP3 inflammasome, where the switch to glycolysis facilitates such inflammasome activation [96]. Blocking glycolysis in microglia has been demonstrated to inhibit Aβ-induced NLRP3 inflammasome activation, thereby reducing neuroinflammation and neurodegeneration [97]. Moreover, mitochondrial dysfunction characterized by increased ROS production in microglia can also trigger NLRP3 inflammasome activation, worsening AD-related pathology [98].

Collectively, the pro-inflammatory cytokines and other mediators produced by systemic inflammation could enter into the brain and induce microglial polarization and then weaken its phagocytosis for the misfolded proteins. The metabolic deficiency of ATP supply accelerates the glycolysis process during the above period of inflammatory response, which can further trigger the over-activation of microglial-mediated neuroinflammation, creating a negative feedback loop of ‘glucose hypometabolism-toxic protein accumulation-neurodegeneration’ [87,99], e.g., causing decreased memory and cognitive dysfunctions in AD patients, and may even lead to abnormal behaviors (Figure 1). Thus, targeting glycolytic metabolic pathways in microglia could potentially provide novel therapeutic approaches to mitigate the harmful effects of abnormal inflammatory microglia and regulate excessive inflammation in brain diseases.

## 4. Anti-Inflammatory Drugs for the Treatment of AD

Clearly, the success rate of anti-AD drug development is extremely low with 99% of candidate drugs discontinued due to lack of clinical benefits. Over 200 anti-AD drugs targeting Aβ and tau therapies have undergone clinical trials, but only two monoclonal antibodies targeting Aβ clearance have received approval from FDA. Aducanumab, one of the monoclonal antibodies targeting Aβ, has shown partial Aβ clearance and cognitive decline slowing effects, but is associated with vasogenic edema. Despite being deemed ineffective in Phase III in March 2019, it was ultimately FDA approved for treating mild AD, leading to significant debate [100]. Lecanemab, another monoclonal antibody against Aβ, targets soluble Aβ oligomers or mature fibrils, demonstrating promising outcomes in inhibiting Aβ and tau pathology with lower clearance activity and fewer side effects compared to aducanumab [101,102]. Consequently, based on the critical role of neuroinflammation in AD pathology, treating neural inflammation from its early stages has emerged as a widely accepted and promising treatment strategy. According to the data from the ClinicalTrials.gov registry, there are about 17% of drugs focusing on inflammation, and in Phase II, these drugs represent 20% of all drugs in development as of the index date of 1 January 2023. The number of anti-inflammatory drugs has been steadily increasing in recent years, with 20 in 2020 (16.5%), 19 in 2021 (15%), and 23 in 2022 (16%) [103]. This section explores investigational drugs that target neuroinflammation and modulate microglial polarization and energy metabolism. These drugs include cyclooxygenase inhibitors, kinase inhibitors, and drugs that regulate microglial polarization and energy metabolic disorder. Table 1 and Table 2 present a comprehensive list of anti-inflammatory small molecule drugs currently in clinical trials, while Table 3 presents the structural formulas of promising drugs in anti-AD research. These tables hope to offer valuable information for advancing drug development in this field.

### 4.1. Non-Steroidal Anti-Inflammatory Drugs (NSAIDs)

NSAIDs are currently the most commonly used antipyretic, analgesic, and anti-inflammatory drugs in clinic. The mechanisms of action of NSAIDs mainly inhibit cyclooxygenase 2 (COX-2) activity to reduce prostaglandin (PG) synthesis. Given the research progress on the relationship between systemic inflammation and microglial activation, NSAIDs also have drawn researchers’ widespread attention in AD treatment for their anti-neuroinflammatory activities, inhibitory effects on Aβ aggregation, and the modulation of γ-secretase [143,144,145]. CHF5074, a non-cyclooxygenase-inhibiting NSAID, has been shown to enhance cognitive performance and reduce intracerebral inflammation by promoting the expression of phagocytic and anti-inflammatory ‘M2’ phenotype markers in young Tg2576 mice [115]. Although CHF5074 (NCT01602393) has been shown to improve cognition and reduce brain inflammation in patients with mild cognitive impairment (MCI), its clinical trial progress has not been updated since the end of the Phase 2 clinical trial in 2014. Another example is azeliragon (NCT02916056), also known as TTP488, which is an inhibitor of the Receptor for Advanced Glycation End-products (RAGE). It is reported that RAGE can react with Aβ to form the RAGE–Aβ interaction, leading to a persistent neuroinflammatory response and the acceleration of AD degeneration [117]. The clinical data from Phase 2b trials of azeliragon suggested that it could slow cognitive decline in mild AD patients [146]. Recent investigations into the underlying mechanisms of azeliragon’s anti-AD properties have shown that it can improve NLRP3-associated inflammation, cell viability, apoptosis, and ROS production by mediating the JAK1/STAT3/NF-κB/IRF3 pathway [147]. Based on promising results of azeliragon from the Phase 2b study, a Phase 3 registration program (STEADFAST and STEADFAST Extension) is being conducted under a Special Protocol Assessment from the FDA in 2021. Although many epidemiological studies have shown that NSAIDs are effective in the treatment of AD, the relative risk of AD is reduced only in people taking long-term NSAIDs at least for more than 24 months [148]. Among them, only indomethacin has shown some slight efficacy in mild to moderate AD in numerous clinical trials of NSAIDs [5]. The protective effects of NSAIDs exhibited in the epidemiological statistics require more than 2 years’ administration of NSAIDs before the onset of AD, suggesting that NSAIDs must be taken very early and at doses that inhibit the advancing inflammation [149]. Other NSAIDs as anti-inflammatory agents under research are furosemide and dendrimers. As reported, furosemide can down-regulate COX-2 and iNOS protein expression to decrease the pro-inflammatory M1 phenotype and increase the anti-inflammatory M2 phenotype of microglia, respectively [150]. A series of low-generation dendrimers were designed and synthesized characterized by both targeting COX-2 inhibition and microglial activation suppression [151].

### 4.2. TLR4 Antagonists

TLR4 is an essential component of the natural immune system, playing an important role in intracellular signaling and neuronal death by regulating the TLR4/MAPKs/NF-κB pathway (Figure 1) to stimulate the production of pro-inflammatory mediators [152]. There is increasing evidence suggesting that the clearance of Aβ by microglia occurs on TLR4 signaling [153]. Studies on TLR4-mutant AD-model mice have shown that they accumulate more Aβ and exhibit greater cognitive deficits as compared to their TLR4-wild-type counterparts [154]. Conversely, TLR4-deficient/knockout AD-mice have demonstrated reduced Aβ deposits and neuroinflammatory factors, improving cognitive abilities [155]. Given the crucial role of TLR4 in AD pathogenesis, it is considered a promising therapeutic target for drug development. TLR4 antagonists such as TAK-242 have shown positive therapeutic effects in various models of peripheral diseases such as inflammatory bowel disease (IBD) by inhibiting the production of inflammatory mediators such as TNF-α, IL-6, and NO [156]. TAK-242, as a specific inhibitor of TLR4, has been found to enhance learning and memory abilities, decrease Aβ deposition, and protect neurons from apoptosis in APP/PS1 mice [118]. Additionally, naloxone has been shown to block TLR4 downstream signaling, leading to NO, TNF-α, and reactive oxygen species, through TLR4 signaling during neuroinflammation [120]. Another promising compound, Gx-50, extracted from Sichuan pepper, has demonstrated potent anti-inflammatory effects against Aβ-triggered microglial over-activation in AD mice by suppressing TLR4-mediated NF-κB/MAPK signaling [119]. Despite the fact that therapeutic targeting of TLR4 in animal models of inflammation or AD has yielded promising results, to date, there are few TLR4 antagonists reported in clinical trials.

### 4.3. p38 Mitogen-Activated Protein Kinase (p38 MAPK) Antagonists

Mitogen-activated protein kinase (MAPK) is a crucial protein family involved in various cellular processes such as cell proliferation, differentiation, inflammation, oxidative stress, and apoptosis in mammals. This family includes p38 MAPK kinase, extracellular signal-regulated kinase (ERK1/2), and c-Jun amino-terminal kinase (JNK)/stress-activated protein kinase (SAPK) [157]. Within the inflammatory signaling pathway, MAPK is primarily regulated by the upstream TLRs pathway, triggered by stimuli like LPS (Figure 1). This leads to the translocation of NF-κb, expression of p38 phosphorylation, and subsequent production of inflammatory factors like TNF-α and IL-6 [158]. Additionally, p38α MAPK kinase was reported to mediate impaired synaptic dysfunction in the hippocampus, causing memory deficits, and to be involved in Aβ production and tau pathology [159]. Based on the central role of p38 MAPK in chronic inflammation, many preclinical or clinical trials for the application of p38 MAPK inhibitors in inflammatory diseases were conducted. VX-745 is an inhibitor that selectively inhibits p38 MAPKα, which was found to be more permeable to the blood–brain barrier (BBB), significantly reducing IL-1β protein levels in the hippocampus and improving memory recognition in aging rats with 22–24 months of age [121]. In a small sample size of a short-term (12 months) Phase IIa clinical trial in early AD patients, VX745 showed good tolerability and adequate drug concentrations in the cerebrospinal fluid (CSF) and a tendency to improve episodic memory [121]. However, in a further long-term (24 months) Phase II clinical trial with a large sample size, VX745 did not show the ability to significantly improve episodic memory in patients with mild AD compared to the placebo group [159,160]. The results obtained from the Phase II clinical trials of VX745 suggest that higher doses and longer duration studies may be necessary to assess its effect on AD progression. Targeted protein degradation offers a unique advantage over gene knockout by selectively degrading proteins with pathological mutations or aberrant post-translational modifications. PRZ-18002, a protein degrader that binds to p38 MAPKα, has been reported to degrade phosphorylated p38 MAPK (p-p38) and mimetic mutants of p38 MAPK in a proteasome-dependent manner, alleviating microglial activation and Aβ deposition. This led to improved spatial memory and learning in a mouse model of AD [122]. Furthermore, natural products and gut microbiota metabolites have been found to inhibit the MAPK signaling pathway with significant anti-neuroinflammatory effects. Urolithin A (UA) and urolithin B (UB), isolated from metabolites of intestinal microorganisms, have shown to reduce NO levels and suppress mRNA levels of pro-inflammatory genes in LPS-treated microglia by inhibiting NF-κB, MAPKs (p38 and ERK1/2), and Akt signaling pathway activation [123].

### 4.4. Microglia Regulators

Cromolyn, a compound used in the clinical treatment of asthma, has been demonstrated to induce neuroprotective activation of microglia and reduce level of aggregation-prone Aβ [127]. Currently, cromolyn is undergoing a phase III trial as a potential agent for early AD treatment (NCT02547818). Furosemide also acted as a modulator of microglia, was found to rescue Aβ-induced neuroinflammation in microglia by regulating microglia polarization to promote anti-inflammatory phenotype transformation [150]. Resveratrol, a natural polyphenol, has the ability to modulate stress signals in microglia [125] leading to the attenuation of cognitive behavioral deficits [126]. Further investigation into the mechanism of action of resveratrol on microglia has revealed its ability to effectively reverse the LPS-induced polarization of microglia from a pro-inflammatory phenotype to an anti-inflammatory phenotype [124]. Research has indicated that PKM2 plays a crucial role in the regulation of energy metabolic disorder of microglia [86,159]. Additionally, TSG and CIAC001 have been identified as compounds with a targeted antagonistic effect on PKM2, demonstrating an anti-neuroinflammatory effect by restoring the PKM2 tetramer levels and inhibiting the nuclear translocation of PKM2 [124,128]. Rui-Yuan Pan discovered that sodium rutin treatment shifts the metabolic program from anaerobic glycolysis to mitochondrial OXPHOS and enhances microglial polarization towards anti-inflammatory phenotypes [130]. Benserazide has been shown to effectively inhibit PKM2, thereby blocking aerobic glycolysis and modulating OXPHOS [129]. While benserazide is a PKM2 inhibitor, currently used in combination with levodopa for PD treatment, its impact on energy metabolic disorder of microglia remains unexplored. Nevertheless, utilizing benserazide as a lead compound for developing novel PKM2 inhibitors to regulate energy metabolic disorder of microglia shows promise in AD treatment [129]. Currently, benzyl hydrazine is used in combination with levodopa for the treatment of Parkinson’s disease, but there is currently no research on how benserazide regulates energy metabolic disorder of microglia. However, we believe that using benserazide as a lead compound for research to develop novel PKM2 inhibitors and regulate energy metabolic disorder of microglia cells is one of the promising directions for treating AD. Rapamycin and salidroside, known mTOR inhibitors, have shown efficacy in AD treatment. However, there is a lack of research on the therapeutic effects of rapamycin in AD through the TREM2/mTOR pathway in regulating energy metabolic disorder of microglia [131,161,162]. Therefore, investigating the potential of rapamycin and salidroside as a research direction for modulating energy metabolic disorder of microglia in microglia is also promising.

### 4.5. Chinese Herbal Polysaccharides

In recent years, with the rapid development of isolation and purification techniques, more and more natural products, especially herbal polysaccharides, play an important role in neurodegenerative diseases such as AD due to their anti-neuroinflammatory and other neuroprotective activities [163]. For example, Ganoderma lucidum polysaccharides can not only promote neural re-generation to improve cognitive impairment in AD transgenic mice but also reduce Aβ levels and tau protein hyperphosphorylation and reduce ultrastructural damage to improve spatial memory in rats [134]. Astragalus polysaccharides can reduce astrocytic and microglial activation and exert biological activities such as antioxidant, anti-neuroinflammatory, and neuroprotective effects [135]. Dendrobium orchid polysaccharide can exert anti-neuroinflammatory effects by inhibiting oxidative stress and reducing the production of free radicals and pro-inflammatory factors such as TNF-α and IL-1β [136]. Furthermore, Astragalus polysaccharide can improve the learning memory ability of zebrafish, a model of AD, by upregulating N-cadherin protein levels and decreasing the phosphorylation of p38 [137]. Chinese medicine polysaccharides are mostly in preclinical studies due to their complex structure compared to small molecule drugs. Fortunately, with the conditional approval of GV-971 [133] by the China Food and Drug Administration in 2019 as a fucoidan for the treatment of mild to moderate AD, the unique advantages of Chinese medicine polysaccharides with mild therapeutic properties and low toxic side effects give them great potential for treatments of neurodegenerative diseases such as AD.

### 4.6. Other Anti-Inflammatory Drugs

In addition to the aforementioned anti-inflammatory drugs including the small molecule inhibitors and regulators, as well as Chinese medicine polysaccharides, there still exist some antibody biologics such as Anakinra, an IL-1β inhibitor [164], and Etanercept, a TNF-α inhibitor [165]. Most of these inhibitors are biomolecules, which are not only costly but have more difficulty to enter the CNS to exert their anti-neuroinflammation through the BBB, severely limiting their possibility in treating neurodegenerative diseases [166]. It is well known that a sharp decrease in acetylcholine occurs during the course of AD, a deficiency of which directly leads to cognitive deficits [167].

Acetylcholine, a neurotransmitter found in the cholinergic nervous system, is released by excited cholinergic neurons. Upon entering the synaptic cleft, acetylcholine can be hydrolyzed by acetylcholinesterase if not bound to receptors, leading to the termination of neurotransmission. Inhibiting acetylcholinesterase can result in the accumulation of acetylcholine, enhancing its action. The cholinergic hypothesis proposes that reduced synthesis of acetylcholine is a key factor in AD [167,168]. Therefore, inhibiting acetylcholinesterase can cause acetylcholine to accumulate, thereby prolonging and enhancing the action of acetylcholine. The cholinergic hypothesis suggests that the main cause of AD is the reduced synthesis of acetylcholine. Therefore, one potential therapeutic strategy for AD is to increase the cholinergic neurotransmitter levels in the brain by inhibiting the biological activity of acetylcholinesterase (AChE) [168]. Therefore, one potential therapeutic approach for AD involves increasing cholinergic neurotransmitter levels by inhibiting acetylcholinesterase activity. Drugs like tacrine, galantamine, donepezil, and cabalatin have been developed to target acetylcholinesterase and alleviate AD symptoms [169]. Due to tacrine’s severe hepatotoxicity, it has been withdrawn from the market [168]. Given its potent anti-AD activity, many new compounds were designed and synthesized by introducing pyrimidine, thiazole, and other heterocycles groups to the parent group of tacrine; one compound (No. 46) was able to inhibit neuroinflammation and aggregation of Aβ with low toxicity and IC50 value of 2 nM of AChE [138]. New compounds, such as compound No. 46, have been designed to inhibit neuroinflammation and Aβ aggregation with low toxicity and high AChE inhibition potency. Currently, acetylcholinesterase inhibitors like donepezil are being investigated in clinical trials, indicating that inhibiting AChE remains a potential strategy for AD treatment. (NCT04661280, NCT05345509, NCT04730635, NCT05525780).

Activation of the NLRP3 inflammasome play a key role in the pathogenesis of AD [45,170,171]. Several compounds, including MCC950, JC-124, CY-09, and inzomelid, have been recognized as inhibitors of NLRP3 with neuroprotective properties. These compounds effectively suppress NLRP3 activation and the production of IL-1β [140,141,142,172].

## 5. Discussion and Outlook

In summary, a large body of evidence suggests that microglial over-activation-mediated neuroinflammation and the energy metabolic disorder of microglia contribute significantly to the pathogenesis of AD, and research on anti-inflammatory drugs for the treatment of AD has also made some progress. In total, 25 anti-inflammatory candidate drugs, including biological preparations and small molecule compounds, have entered clinical trials, ranking second only to neurotransmitter receptor drugs as of 1 January 2023 [103]. Among them, masitinib and NE3107 have entered Phase III clinical trials, emphasizing that suppressing neuroinflammation has been considered as a potential therapeutic strategy for AD. Clearly, microglia are crucial mediators and effectors in the pathology of AD, but a slew of mysteries surrounding the interactions between microglia and AD remain unsolved. The early stage of microglial activation-mediated neurodegeneration has been linked to ‘M1’ phenotype-associated inflammatory factors such as TNF-α and IL-1β (Figure 1). In this stage, microglia are activated to endocytose pathological Aβ and tau but with cytotoxic effects, and the inefficiency of ATP supply accelerates the glycolysis process of microglia followed by continued microglial overactivation (Figure 1). In the later stage of microglial activation, the ‘M1’ phenotype fails to endocytose pathological Aβ and tau and their deposition return contributes to inflammatory activation and energy metabolism disorder of microglia. On the contrary, anti-inflammatory factors secreted by ‘M1’ phenotype microglia, such as IL-4 and IL-10, and inhibition of certain receptors, such as TREM2, PKM2, and NLRP3, aid in the restoration of learning and memory deficits in AD via various signaling pathways and mechanisms (Figure 1). The above double-edged sword mechanism of microglia regulation between ’protective immune effector‘ and ’harmful inducer-mediated neuroinflammation in AD‘ might constrain the development of anti-inflammatory drugs in AD treatment. Notably, long-term clinical use of NSAIDs has been epidemiologically shown to significantly reduce the risk of developing AD, suggesting the vital role of inflammation in AD pathology and the beneficial effect of anti-inflammatory agents for AD prevention and treatment. Although the short duration of clinical trials on NSAIDs for AD patients failed to exhibit direct evidence of clinical efficacy, as the research advances on the pathogenesis of microglia-mediated neuroinflammation in AD and given the continuous improvement of clinical trial design schemes considering the difference between the short and long duration, further progress will be made in the treatment of AD with anti-inflammatory drugs.

## Figures and Tables

**Table 1:** AD anti-inflammation drug in clinical development (https://classic.clinicaltrials.gov/ (access on 6 March 2024)).

Agent	Type	CADRO Target	Mechanism of Action	Clinical Trial NCT#
Masitinib (Phase 3)	small molecule	Inflammation	Tyrosine kinase inhibitor exhibits neuroprotection via inhibition of mast cell and microglia/macrophage activity	NCT05564169
NE3107 (Phase 3)	small molecule	Inflammation	Beta-androstenetriol with anti-inflammatory and insulin signaling effects via ERK 1 and 2	NCT04669028
Baricitinib (Phase 2)	small molecule	Inflammation	Janus kinase (JAK) inhibitor	NCT05189106
Dasatinib + Quercetin (Phase 2)	small molecule	Inflammation	Dasatinib induces apoptosis in senescent cells to allow their removal; quercetin is a flavonoid	NCT04063124
				NCT04685590
				NCT04785300
				NCT05422885
L-Serine (Phase 2)	small molecule	Inflammation	Naturally occurring dietary amino acid; inhibits toxic misfolding	NCT03062449
Lenalidomide (Phase 2)	small molecule	Inflammation	Immunomodulator	NCT04032626
Montelukast (Phase 2)	small molecule	Inflammation	Leukotriene receptor antagonist (LTRA); anti-inflammatory effects	NCT03402503
Senicapoc (Phase 2)	small molecule	Inflammation	Calcium-activated potassium channel inhibitor	NCT04804241
Valacyclovir (Phase 2)	small molecule	Inflammation	Anti-viral against HSV-1 and −2; reduces vira-related “seeding” of ABP deposition	NCT03282916
Salsalate (Phase 1)	small molecule	Inflammation	Non-steroidal anti-inflammatory (NSAID)	NCT03277573
Emtricitabine (Phase 1)	small molecule	Inflammation	Nucleoside reverse transcriptase inhibitor (NRTI)	NCT04500847

**Table 2:** AD anti-inflammation drug mechanism (https://classic.clinicaltrials.gov/ (access on 6 March 2024)).

Drug Name	Chemical Structures	Category	Compound Effects
Masitinib	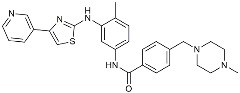	Tyrosine kinase inhibitor	Downregulated proinflammatory cytokines. Induced neuroprotection [104].
NE3107	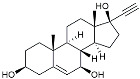	NF-κB inhibitor	Decreased activated microglia, Aβ [105].
Baricitinib	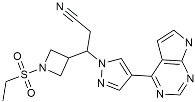	JAK inhibitor	Blocked intracellular delivery of cytokines via JAK-STAT [106].
Dasatinib + Quercetin	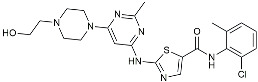	Tyrosine kinase inhibitor	Alleviated neurodegeneration in AD [107].
	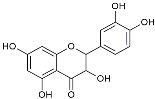	PI3K/Akt inhibitor	
L-Serine	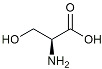	mTOR inhibitor	Autophagic clearance of Aβ [108].
Lenalidomide	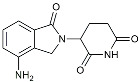	Immunomodulator	Decreased the expression of TNFα, IL-6, IL-8. Increased the expression of anti-inflammatory cytokines [109].
Montelukast	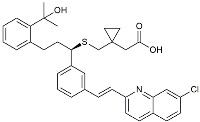	Cytochrome P-450 Enzyme Inducers	Increased expression of P450 enzymes [110].
Senicapoc	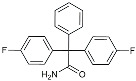	K_Ca_3.1 inhibitor	Regulated microglia polarization [111].
Valacyclovir	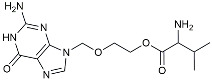	Anti-virus	Reduced accumulation of Aβ and p-tau [112].
Salsalate	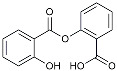	NASID	Inhibited inflammatory mediators [113].
Emtricitabine	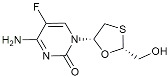	Anti-virus	Suppressed neuroinflammation [114].

**Table 3:** Promising drugs for treating AD by inhibiting inflammation and regulating microglia polarization and energy metabolic disorder.

Drug Name	Chemical Structures	Category	Compound Effects
CHF5074	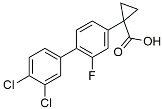		Improved cognition. Reduced brain inflammation [115].
Indomethacin	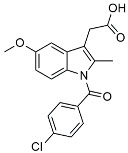	Cox-2 inhibitor	Inhibited inflammatory mediators released from microglia [116].
Furosemide	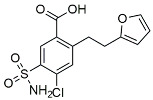		Inhibited protein expression of COX-2,iNOS.
Azeliragon	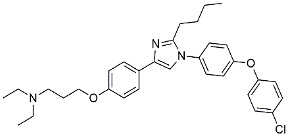		Mediated the JAK1/STAT3/NF-κB/IRF3 pathway [117].
TAK242	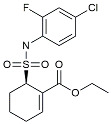	TLR4 antagonists	Reduced Aβ deposition [118].
Gx-50	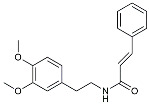		Suppressed of TLR4-mediated NF-κB/MAPK signaling [119].
Naloxone	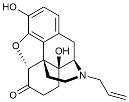		Inhibited reactive oxygen species production [120].
VX745	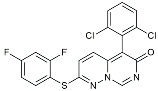	p38-MAPK antagonists	Reduced IL-1β protein levels in the hippocampus [121].
PRZ-18002	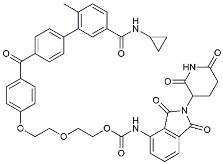		Degraded phosphorylated p38 MAPK (p-p38) [122].
			Alleviated microglial activation and Aβ deposition [122].
Urolithin	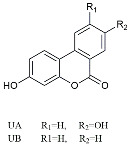		Reduced NO levels and suppressed pro-inflammatory genes [123].
TSG	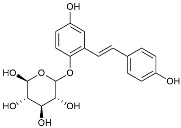	Microglia regulators	Inhibited PKM2 to adjust microglia polarization [124].
Resveratrol	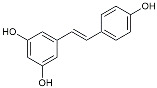		Adjusted microglia polarization [124,125,126].
Cromolyn	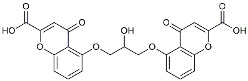		Induced neuroprotective microglial activation [127].
CIAC001	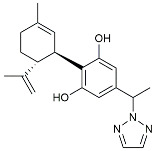	PKM2 inhibitor	Ameliorated morphine-induced addiction through anti-neuroinflammation [128].
Benserazide	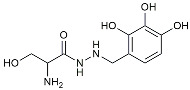		Inhibited PKM2, thereby blocking aerobic glycolysis and modulating OXPHOS [129].
Rutin	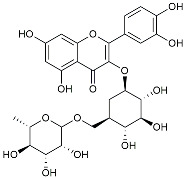		Promoted a metabolic switch from anaerobic glycolysis to mitochondrial OXPHOS [130].
Rapamycin	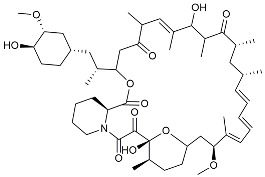	mTOR inhibitor	Activated mitophagy and alleviated cognitive impairment [131].
Salidroside	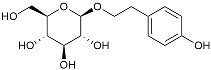		Inhibited Aβ deposit. Anti-inflammation [132].
GV-971	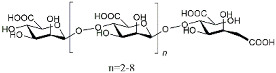	Chinese herbal polysaccharides	Remodeled gut microbiota to inhibit AD progression [133].
Ganoderma lucidum polysaccharides	/		Reduced Aβ levels and tau protein hyperphosphorylation [134].
Astragalus polysaccharides	/		Reduced astrocytic and microglial activation [135].
Dendrobium orchid polysaccharide	/		Inhibited oxidative stress [136].
Astragalus polysaccharide	/		Decreased the phosphorylation of p38 MAPK [137].
Tacrine	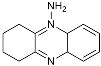	AChE inhibitor	Inhibited acetylcholinesterase activity [138].
The derivative of tacrine	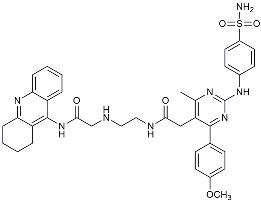		
Donepezil	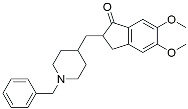		
Dapansutrile	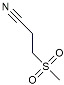	NLRP3 Inhibitor	Inhibited the associated NLRP3 inflammatory response [120].
MC950	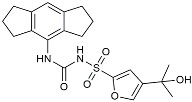		Reduced Aβ deposition and associated neurotoxicity [139].
JC124	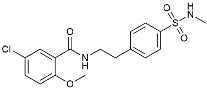		Reduced CAA, microgliosis and oxidative stress [140].
CY-09	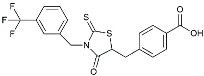		Inhibited the associated NLRP3 inflammatory response [141].
Inzomelid	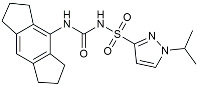		Inhibited the associated NLRP3 inflammatory response [142].

## Abbreviations

Aβ: Amyloid beta, AD: Alzheimer’s disease, APP: Amyloid protein precursor, ABPs: beta-amyloid plaques, AChE: acetylcholinesterase, CNS: Central nervous system, COX: Cyclooxygenase, DAMPs: Damage-associated molecular patterns, IL-1β: Interleukin 1 beta, IL-6: interleukin 6, LPS: Lipopolysaccharides, mTOR: Mammalian target of rapamycin, MAPK: mitogen-activated protein kinase, NLRP3: NLR: family pyrin domain containing 3, NSAIDs: Non-steroidal anti-inflammatory drugs, OXPHOS: oxidative phosphorylation, PAMPs: Pathogen-associated molecular patterns, PRRs: pattern recognition receptors, PKM2: pyruvate kinase M2, SPs: senile plaques, TREM2: triggering receptor expressed on myeloid cells 2, TGF-β: transforming growth factor-β, TLRs: Toll-like receptors, TNF-α: Tumor necrosis factor alpha, TLR4: Toll-like receptor 4, NFTs: neurofibrillary tangles.
